# Accounting for Missing Data in Public Health Research Using a Synthesis of Statistical and Mathematical Models

**DOI:** 10.1136/jech-2025-224750

**Published:** 2026-01-09

**Authors:** Paul N Zivich, Bonnie E Shook-Sa, Stephen R Cole, Eric T Lofgren, Jessie K Edwards

**Affiliations:** 1 Department of Epidemiology, Gillings School of Global Public Health, University of North Carolina at Chapel Hill, Chapel Hill, NC, USA; 2 Department of Biostatistics, Gillings School of Global Public Health, University of North Carolina at Chapel Hill, Chapel Hill, NC, USA; 3 Nuffield Department of Population Health, University of Oxford, Oxford, UK; 4 Paul G. Allen School for Global Health, Washington State University, Pullman, WA, USA

## Abstract

**Introduction:**

Accounting for missing data by imputing or weighting conditional on covariates relies on the variable with missingness being observed at least some of the time for all unique covariate values. This requirement is referred to as positivity and positivity violations can result in bias. Here, we review a novel approach to addressing positivity violations in the context of systolic blood pressure.

**Methods:**

To illustrate the proposed approach, we estimate the mean systolic blood pressure among children and adolescents aged 2-17 years old in the United States using data from the 2017-2018 National Health and Nutrition Examination Survey (NHANES). As blood pressure was not measured for those aged 2-7, there exists a positivity violation by design. Using a recently proposed synthesis of statistical and mathematical models, we integrate external information with NHANES to address our motivating question.

**Results:**

With the synthesis model, the estimated mean systolic blood pressure was 100.5 (95% confidence interval: 99.9, 101.0), which is notably lower than either a complete-case analysis or extrapolation from a statistical model. The synthesis results were supported by a diagnostic comparing the performance of the mathematical model in the positive region.

**Discussion:**

Positivity violations pose a threat to quantitative medical research, and standard approaches to addressing nonpositivity rely on restrictive untestable assumptions. Using a synthesis model, like the one detailed here, offers a viable alternative.

## Introduction

Suppose we are interested in estimating the mean systolic blood pressure (SBP) among children and adolescents aged 2-17 in the United States (US) between 2017-2018. To estimate this mean, we use data from the National Health and Nutrition Examination Survey (NHANES), a nationally representative survey of children in the US.^1^ However, SBP measurements are partially missing. While we could restrict our data to those with measured SBP (i.e., conduct a compete-case analysis), this approach may be biased when there is a variable predictive of both SBP and missingness of SBP.^2^ Instead, methods like weighting and imputation can be used to correct for missing outcome data.^3^ In absence of strong parametric modeling assumptions, these methods assume that SBP is measured for at least some people for each unique value of the measured variables. For example, if missingness depends on age (which is related to SBP) then these methods assume that SBP has a non-zero probability of being measured for each age. This assumption is referred to as positivity and can lead to bias when it is violated.^2,4–6^ Existing approaches to address nonpositivity require modifying the scientific question or extrapolating from a statistical model. In recent work, Zivich and colleagues introduced a new approach that avoids the issues of these existing approaches.^7,8^ Here, we review the problem posed by nonpositivity and illustrate this novel alternative with publicly available data and corresponding code in R and Python to replicate the analyses.^9^

## Methods

Here, we consider the following variables collected by NHANES. The variable of interest, SBP (mm Hg), was measured up to three times. In this analysis, we took the mean of the available SBP measurements and SBP was set to missing if fewer than two measurements were available. Sampling weights were applied for inference to the US population. Additional covariates included age (years), height (centimeters), weight (kilograms), and gender (male, female).

In the NHANES data, SBP was missing for 44% of children. Unless SBP and the probability of missing SBP are independent, a complete-case analysis (i.e., restricting to only those with measured SBP) can be substantially biased.^2^ To help make these ideas more precise, we introduce some notation. Let *X* be age, *Y* be SBP, and *R* = 1 indicate that SBP was observed. Our parameter of interest, the mean SBP for children and adolescents, can be expressed as *μ* = *E*[*Y*], where *E*[⋅] is the expected value function. However, SBP is only observed for those with *R* = 1. For a complete-case analysis to be unbiased, SBP and missingness must be marginally independent or exchangeable,^3^ which can be expressed as *E*[*Y*] = *E*[*Y* | *R* = 1] and indicates that those with a measured SBP are a random sample of all children and adolescents. This assumption would be valid if NHANES randomly determined which participants had their SBP measured. However, this assumption is not consistent with the NHANES design.

With missing data, a marginal exchangeability assumption is often unreasonable, as investigators rarely have control over non-response. However, weaker assumptions regarding the missing data mechanism can be made. Rather than *marginal* exchangeability, we can instead assume *conditional* exchangeability. For didactic purposes, consider if missingness was independent of SBP conditional on age. The conditional exchangeability assumption can then be expressed as *E*[*Y* | *X* = *x*] = *E*[*Y* | *X* = *x, R* = 1] for all ages *x* from 2-17. This assumption states that those with a measured SBP are a random sample *within each stratum of age* from 2 to 17, allowing those with a measured SBP to ‘stand-in’ for those with an unmeasured SBP that are the same age. Implicitly, this exchangeability assumption comes with the positivity assumption that SBP has a non-zero probability of being measured for each unique age in our population.^4,10^ This assumption can be written as Pr(*R* = 1 | *X* = *x*) > 0 for all ages *x* from 2-17. To illustrate why positivity is important, note that if everyone of a given age does not have a measured SBP, then there would be no one with a measured SBP to stand-in for them.

Given conditional exchangeability with positivity, we can express the parameter of interest, *μ*, in terms of the data we observed. Specifically, we can show that

$$E[Y]=\sum_{x}E[Y|X=x,R=1]\mathrm{Pr}(X=x)$$

following from the law of total expectation over all ages *x* from 2-17 and exchangeability with positivity. As the right-hand side is expressed in terms of the observed data, this result provides a recipe for estimating *μ*. Specifically, it motivates a direct maximum likelihood estimator, also referred to as g-computation or imputation.^2,3^ To apply this method, one first estimates a regression model for SBP given age among all observations with complete data and the NHANES sampling weights. Then using this estimated model, one predicts SBP given age for *all* children in the data set. Effectively, this approach fills in the missing values for all observations. The mean SBP is then estimated by taking the sample-weighted average of the predicted SBP.

### Nonpositivity

Positivity is violated in our example, as NHANES did not measure SBP for any participants <8 years old. To reiterate why nonpositivity is an issue, suppose we considered the following model for SBP given age

$$E[Y|X,R=1;\beta ]=\beta_{2}I(X=2)+\beta_{3}I(X=3)+\cdots +\beta_{17}I(X=17)$$

where *I*(⋅) is the indicator function. In this model, there is a unique coefficient for each age (i.e., the model is saturated), so no parametric modeling assumptions are imposed. Nonpositivity means that there is no one with SBP measured for ages <8, so the coefficients from *β*_2_ through *β*_7_ cannot be estimated using the NHANES data alone.

### Existing Approaches

To proceed when there is nonpositivity, we could modify the research question. Here, we could consider estimating the mean SBP in children aged 8-17 instead, represented mathematically as *E*[*Y* ∣ *X* ≥ 8]. While valid, this revision addresses a different research question and thus may limit the utility of this data analysis if interest is really in the 2-17 age range. Instead, consider another approach to address the original research question. One could incorporate structural or parametric assumptions into a statistical model and use that model to extrapolate to ages with unmeasured SBP. For example, we might consider the following linear model

$$E[Y|X,R=1,;\beta ]=\beta_{0}+\beta_{1}X$$

which imposes a linear relationship between age and SBP. This model allows us to fill-in SBP values of those aged 2-7 using a model fit to those aged 8-17. Use of this regression model requires two important assumptions. First, the model assumes that there is a linear relationship between SBP and age among those 8-17. This assumption is possible to assess and relax with the available data. Second, the use of this model assumes that the linear relationship extends to those aged 2-7. In other words, the model fit to those aged 8-17 is used to extrapolate to those aged 2-7. The assumption licensing this extrapolation cannot be assessed with the available NHANES data.

### Proposed Approach

To avoid the restrictive modeling assumptions of the extrapolation approach while still addressing the motivating question, we review a method based on a synthesis of statistical and mathematical models.^7^ To motivate this approach, first note that we can divide the data into two parts: one where positivity is met and one where positivity is not met. As shorthand, let X* = 1 if a NHANES participant’s age was between 8-17 and X* = 0 otherwise. Using this additional notation and the idea of dividing the data regarding whether positivity is met, we can rewrite *μ* as

$$E[Y]=E[Y|X^{*}=1]\mathrm{Pr}(X^{*}=1)+E[Y|X^{*}=0]\mathrm{Pr}(X^{*}=0)$$

which is a weighted average of the means in the positive and nonpositive regions. Since X* is observed for all participants, Pr(X* = 1) and Pr(X* = 0) can be directly estimated from the data. Therefore, how *E*[*Y* | X* = 1] and *E*[*Y* | X* = 0] can be learned is the remaining task. The core idea of the synthesis model is to rely on a statistical model to address missingness in the region with positivity (i.e., *E*[*Y* | X* = 1]) and use a mathematical model to fill-in missing values using external information in the nonpositive region (i.e., *E*[*Y* | X* = 0]).

Starting with *E*[*Y* | X* = 1], recall that this is the region with positivity which means standard statistical methods for missing data can be applied. Specifically, we can rely on the previous conditional exchangeability with positivity assumptions limited to those aged 8-17. These assumptions allow us to use the previously described g-computation procedure limited to those aged 8-17. Here, we use the following saturated model

$$E[Y|X,X^{*}=1,R=1,\beta ]=\beta_{8}I(X=8)+\beta_{9}I(X=9)+\cdots +\beta_{17}I(X=17)$$

which places no parametric constraints on the relationship between age and SBP for those aged 8-17. Unlike the extrapolation approach for dealing with nonpositivity, this statistical model does not require there to be a linear relationship between age and SBP for those ages 8-17.

Now, we turn attention to *E*[*Y* | X* = 0]. To reiterate, SBP was never measured for X* = 0 in the data. Therefore, we need to look outside the available NHANES data to make progress. To start, one might consider plugging in extreme but plausible values for the mean among 2-7 year olds (e.g., 70 to 120 mm Hg).^8^ Plugging in these values provides a range of plausible values for *μ*, often referred to as bounds.^11^ These bounds provide a simple sensitivity analysis that only relies on the assumption that the true *E*[*Y* | X* = 0] lies somewhere within the chosen range. How *μ* changes across the range of plausible values can also be plotted to further explore the bounds.

When additional external information is available, we can move beyond a sensitivity analysis and instead build a mathematical model to fill-in the missing values. Here, values for those aged 2-7 are imputed based on published SBP distributions for US children and adolescents.^12^ The imputed values from the mathematical model come from random draws of age, gender, and height percentile specific SBP distributions. Here, we assume normal distributions under the assumption that the mean is equal to the median and the standard deviation is approximated from the 90^th^ percentile. Use of this mathematical model to fill-in missing values of SBP is justified by the assumption that the given external information is accurate for our context. Here, accurate external information for our context means that the source population for the published SBP descriptive statistics and NHANES population are similar for all other predictors of SBP besides age, gender, and height. This assumption would be violated, for example, if weight were distributed differently across data sources. As NHANES and the published SBP distributions are both intended to be representative of the US population, we expect any differences to be small.

To apply the synthesis model while also accounting for variability in both the statistical and mathematical models, we use a resampling procedure that incorporates the NHANES sample weights. The algorithm for this procedure is described in Appendix 1. Briefly, the NHANES data is resampled with replacement. The parameters of the statistical model are re-estimated and used to estimate *E*[*Y* | X* = 1]. Similarly, new imputed values are drawn from the mathematical model in order to estimate *E*[*Y* | X* = 0]. These values are then used to estimate *μ*. This process is repeated a large number of times (i.e., 10,000), and the different estimates of *μ* are summarized by the median as the point estimate, and 2.5^th^ and 97.5^th^ percentiles as the 95% confidence interval (CI). This procedure accounts for the sampling uncertainty in NHANES as well as the uncertainty in the mathematical model parameters. However, this procedure does not account for clustering in the NHANES design, so this procedure likely is an underestimate of the uncertainty.

As mentioned, the validity of this approach is premised on the mathematical model being built from accurate external information. While there are substantive reasons to believe this assumption to be reasonable, the external information allows us to indirectly check the validity of this assumption. Here, the external information also included SBP distributions for those aged 8-17. Using this additional information, we compare the age-specific SBP values for those aged 8-17 between the statistical and mathematical models. To incorporate sources of uncertainty, the resampling procedure is used to compare the difference between the age-specific SBP averages by model. While we believe such a check to have utility, it should be recognized that this diagnostic cannot assess the applicability of the mathematical model for the nonpositive region.

In practice, researchers may wish to incorporate more than one variable in their imputation model. For example, one could instead assume exchangeability conditional on age, gender, height, and weight within the positive region. In Appendix 2, we show how the synthesis approach can be applied with a parametric statistical model to account for these variables in the positive region. As alluded to earlier, use of parametric statistical models relies on the assumption that the model is correctly specified. When this assumption is not met, estimates of *μ* may be biased. In Appendix 3, we describe an augmented inverse probability weighting estimator for synthesis models that relies on less restrictive parametric modeling assumptions.

### Research Ethics

Ethical approval for this study was not required as this was a secondary data analysis of de-identified data and thus not considered to be human subject research under United States federal regulation 45 CFR 46.102(e)(1). The design of NHANES was originally approved by the National Center for Health Statistics Ethics Review Board.

## Results

**Figure 1:**
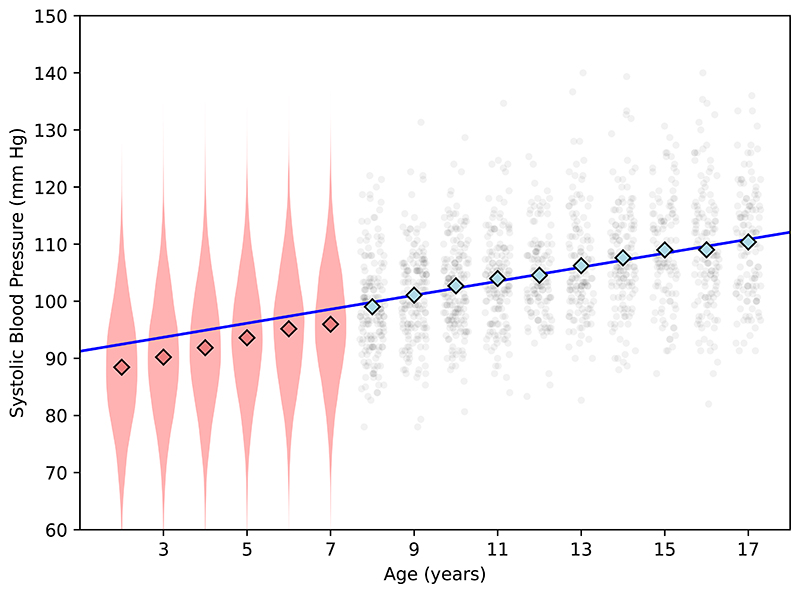
Systolic blood pressure by age from observations in the 2017-2018 National Health and Nutrition Examination Survey and projections from the mathematical model Caption: Gray dots indicate the observed systolic blood pressure values by age (uniformly jittered for visualization) from NHANES. Systolic blood pressure was not measured for those younger than 8 years old. The blue diamonds indicate the age-specific means. The blue line indicates a linear model fit to the observed data and projected for those under 8. The red shaded region indicates the distributions of 20,000 simulated observations from the mathematical model, with the red diamonds indicating the corresponding mean.

**Figure 2:**
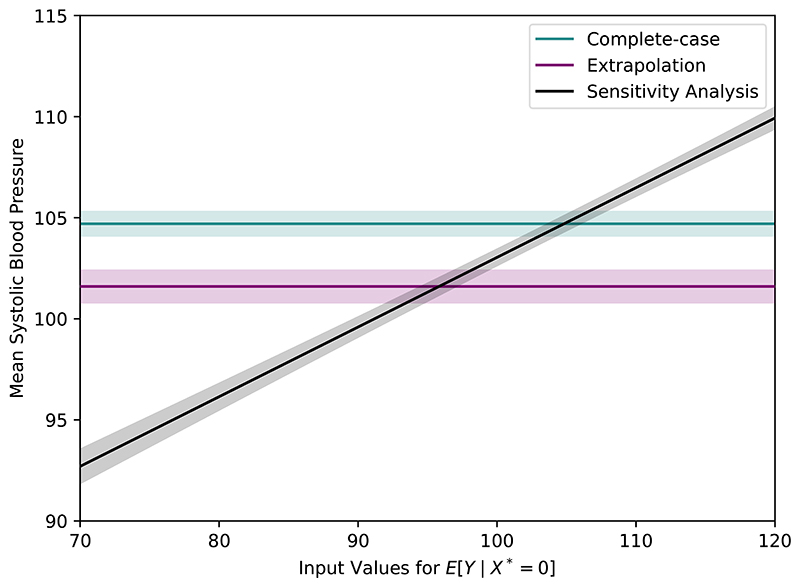
Sensitivity analysis for non-positivity on the mean systolic blood pressure in the 2017-2018 National Health and Nutrition Examination Survey Caption: The black line indicates how the mean systolic blood pressure for children aged 2-17 (y-axis) varies under differing input values for the mean in the non-positive region (i.e., children aged 2-8) across the range 70 to 120 mm Hg (x-axis). The colored horizontal lines represent the results from the complete-case and extrapolation analyses and show how they relate to the sensitivity analysis. Shaded regions denote the 95% confidence intervals.

Those aged 2-7 made up 34% of the NHANES weighted sample (Appendix Table 1). Among those aged 8-17, SBP was missing for 8%. In a complete-case analysis, the estimated mean SBP was 104.7 (95% CI: 104.1, 105.3). These results are questionable given what is known about the missing data and the relation between age and SBP. This estimate is more reflective of the mean SBP among those aged 8-17. With a linear extrapolation from the statistical model (Figure 1), the estimated mean SBP among children and adolescents was noticeably lower (mean: 101.6, 95% CI: 100.8, 102.4). The difference between the complete-case analysis and extrapolation aligns with expectations, as those under 8 years old are expected to have lower SBP. If the mean SBP for those aged 2-7 was bound between 70-120, the bounds for overall mean SBP would have ranged from 92.7 to 109.9 (95% CI: 91.9, 110.5). Figure 2 shows how *μ* varies across this range. With a mathematical model, the estimated mean SBP was lower than the extrapolation approach, at 100.5 (95% CI: 99.9, 101.0). This difference arises from the linear model imputing higher mean SBP for lower ages relative to the mathematical model (Figure 1). While the extrapolation and synthesis model results may not appear substantially different, the observed 1.1 mm Hg difference is 2.9 times the estimated standard error of the extrapolation approach. When incorporating gender, height, and weight into the outcome model, results for the synthesis model were unchanged (Appendix 2). However, the extrapolation approach results moved closer to the synthesis approach. Results did not substantially change further when using an augmented inverse probability weighting estimator (Appendix 3).

**Figure 3:**
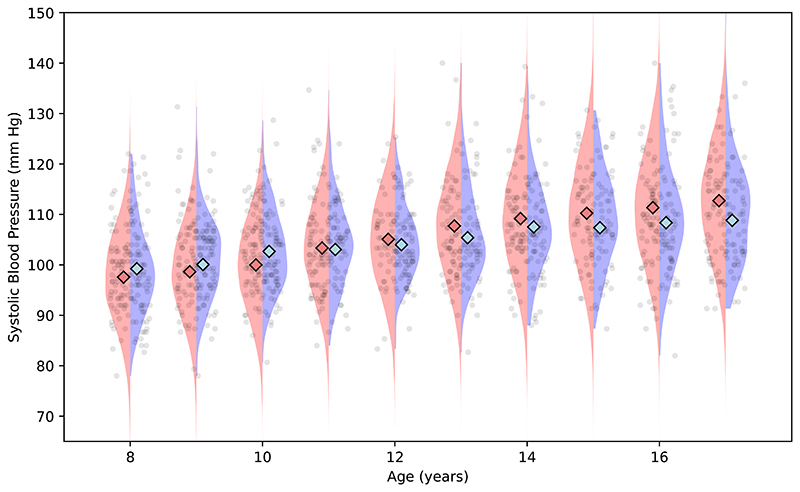
Comparison of statistical and mathematical model values for systolic blood pressure by age in the positive region. Caption: Gray dots indicate the observed systolic blood pressure values by age (uniformly jittered for visualization) from NHANES. Blue diamonds indicate age-specific means from the statistical model. The red shaded region indicates the distributions of 20,000 simulated observations at each age from the mathematical model, with the red diamonds indicating the corresponding mean.

**Figure 4:**
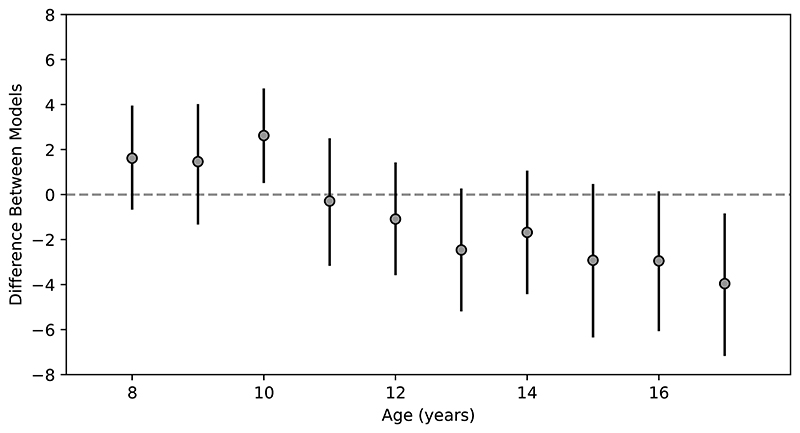
Differences between mean systolic blood pressure comparing statistical versus mathematical models and corresponding 95% confidence intervals. Caption: Circles indicate the difference between the statistical model and mathematical model for each of the means among those in the positive region. Vertical lines indicate the corresponding 95% confidence intervals.

When examining the validity of the mathematical model using the aforementioned diagnostic, the distribution of predicted SBP from the mathematical model overlaps with the observed SBP values for each age (Figure 3). When comparing the differences between means and incorporating uncertainty, the estimated means from either approach were reasonably close to zero (Figure 4). However, the statistical model results seemed to be consistently smaller than the mathematical model for those aged 15-17. Altogether these results provide some support for the validity of the mathematical model in the positive region, which helps to support the belief that the mathematical model is also reasonable for the uncheckable nonpositive region.

## Discussion

Nonpositivity threatens our ability to address important public health questions.^6^ While described here in the context of missing data, the positivity assumption also appears when addressing other biases, like confounding and selection bias.^4^ Historically, nonpositivity has pushed public health and medical researchers to modify their questions or make restrictive, untestable assumptions.^7^ Here, we illustrated an alternative in the context of missing data on SBP using publicly available data from NHANES. The proposed approach is based on integrating the available data with external information through a synthesis of statistical and mathematical models. Importantly, this approach does not require modifying the motivating question or reliance on restrictive modeling assumptions, unlike competing methods.

There are a number of limitations to our analysis and the NHANES example provided here should be viewed only as illustrative. Regarding variance estimation, the approaches used here did not account for the use of cluster sampling in NHANES, so the variance may be underestimated. Extending the resampling algorithm to include additional complexities, like clustering, is an area for future research. Regarding the mathematical model, a fairly simple model was used here to directly impute SBP values. Simple models like this may not always be feasible. Instead, intermediate processes may need to be modeled. For example, imputing missing SBP data for adults on hypertensive medication with a mathematical model may instead involve modeling active drug concentrations and subsequent physiological responses.^13^ Designing and building complex mathematical models should follow best practices,^14–16^ and evaluate diagnostic procedures whenever possible. Future work should illustrate use of these more advanced models. Finally, non-positivity may occur for more than a single variable. While the essential concepts of synthesis modeling still apply, illustrating their use in these contexts remains needed.

## Supplementary Material

Supplement

## Data Availability

Data and code to replicate the example are provided on GitHub (https://github.com/pzivich/publications-code).

## Key Messages

### What is Already Known on this Topic

- Positivity is an important underlying assumption for many methods to address systematic errors, or biases, in epidemiologic research.
- When there is nonpositivity, epidemiologists have had to modify their scientific questions or rely on strong parametric modeling assumptions to extrapolate.

### What this Study Adds

- A recently proposed method to address positivity violations based on a synthesis of statistical and mathematical models is reviewed and illustrated in the context of systolic blood pressure, missing data, and nonpositivity.

### How this Study Might Affect Research, Practice, or Policy

- The method reviewed in this article offers a way forward when the positivity assumption is not met which does not require modifying the motivating epidemiologic question or having to rely on extrapolation.

## References

[1] NHANES Questionnaires, Datasets, and Related Documentation.

[2] Cole SR, Zivich PN, Edwards JK (2023). Missing Outcome Data in Epidemiologic Studies. American Journal of Epidemiology.

[3] Vansteelandt S, Carpenter J, Kenward MG (2010). Analysis of incomplete data using inverse probability weighting and doubly robust estimators. Methodology: European Journal of Research Methods for the Behavioral and Social Sciences.

[4] Zivich PN, Cole SR, Westreich D Positivity: Identifiability and Estimability. arXiv.

[5] Petersen ML, Porter KE, Gruber S, Wang Y, van der Laan MJ (2012). Diagnosing and responding to violations in the positivity assumption. Stat Methods Med Res.

[6] Westreich D, Cole SR (2010). Invited commentary: positivity in practice. American Journal of Epidemiology.

[7] Zivich PN, Edwards JK, Lofgren ET, Cole SR, Shook-Sa BE, Lessler J (2024). Transportability without positivity: a synthesis of statistical and simulation modeling. Epidemiology.

[8] Zivich PN, Edwards JK, Shook-Sa BE, Lofgren ET, Lessler J, Cole SR Synthesis estimators for transportability with positivity violations by a continuous covariate. Journal of the Royal Statistical Society Series A: Statistics in Society.

[9] Zivich P pzivich/publications-code: v2025.05.03.

[10] Hernán MA, Robins JM (2006). Estimating causal effects from epidemiological data. Journal of Epidemiology and Community Health.

[11] Manski CF (2005). Partial identification with missing data: concepts and findings. International Journal of Approximate Reasoning.

[12] Flynn JT, Kaelber DC, Baker-Smith CM (2017). Clinical Practice Guideline for Screening and Management of High Blood Pressure in Children and Adolescents. Pediatrics.

[13] Heo YA, Holford N, Kim Y, Son M, Park K (2016). Quantitative model for the blood pressure-lowering interaction of valsartan and amlodipine. British Journal of Clinical Pharmacology.

[14] Roberts M, Russell LB, Paltiel AD, Chambers M, McEwan P, Krahn M (2012). Conceptualizing a Model: A Report of the ISPOR-SMDM Modeling Good Research Practices Task Force–2. Med Decis Making.

[15] Krijkamp EM, Alarid-Escudero F, Enns EA, Jalal HJ, Hunink MGM, Pechlivanoglou P (2018). Microsimulation modeling for health decision sciences using R: a tutorial. Med Decis Making.

[16] Slayton RB, O’Hagan JJ, Barnes S (2020). Modeling Infectious Diseases in Healthcare Network (MInD-Healthcare) Framework for Describing and Reporting Multidrug-resistant Organism and Healthcare-Associated Infections Agent-based Modeling Methods. Clinical Infectious Diseases.

