# Supplementary material for: Accounting for Missing Data in Public Health Research Using a Synthesis of Statistical and Mathematical Models
Source: J Epidemiol Community Health. Author manuscript; Available in PMC 2026 May 1. (PMC7618619; doi:10.1136/jech-2025-224750)
Supplement: Supplement [file EMS211453-supplement-Supplement.pdf]

Accounting for Missing Data in Public Health Research Using a Synthesis of Statistical and Mathematical Models

Appendix

Appendix 1: Resampling Algorithm for Uncertainty 2

Appendix 2: Extended Conditional Exchangeability 3

Appendix 3: Extended Conditional Exchangeability 5

Appendix 3.1: Inverse Probability Weighting 5

Appendix 3.2: Augmented Inverse Probability Weighting 6

Appendix 3.3: Results 6

Appendix Table 1 7

References 8

Appendix 1: Resampling Algorithm for Uncertainty

To incorporate the uncertainty in both estimation of the statistical model parameters and the chosen distributions for the mathematical model, the following resampling algorithm is used.

1. For k from 1 to K:
    a. Resample with replacement n observations from the full NHANES data set.
    b. Divide the resampled data into the positive and non-positivity regions.
    c. With the resampled NHANES data for the positive region, fit the specified statistical model using observations without missing data on the outcome. From the fitted model, predict the outcome for all observations in the positive region, regardless of whether observations had missing outcomes.
    d. With the resampled data for the non-positive region, randomly draw values for the imputed outcomes from the specified mathematical model.
    e. Pool data from the positive and non-positive regions and take the mean of the imputed outcomes. If the data has sample weights, like NHANES, take the sample-weighted mean. Save this mean value as $\hat{\mu}_k$.
2. Summarize the collection of $(\hat{\mu}_1, \ldots, \hat{\mu}_K)$. The point estimate can be obtained by taking the median. Two-sided 95% confidence intervals can be obtained by taking the 2.5^{th} and 97.5^{th} percentiles. The values can also be plotted using a histogram to visualize the distribution.

For intuition behind this procedure, note that this procedure involves re-estimating the statistical model parameters using data resampled with replacement, like the nonparametric bootstrap.[1] Similarly, the mathematical model is used to draw values from the chosen distribution repeatedly. This provides a collection of estimates which are then jointly summarized.

When computing the confidence intervals for the bounds or sensitivity analysis, the mathematical model no longer contributes to the uncertainty since it is set to a fixed value. A resampling procedure (i.e., bootstrap) can be used for inference in these settings, or one can use other appropriate statistical variance estimation procedures (e.g., the empirical sandwich variance estimator).[2]

Appendix 2: Extended Conditional Exchangeability

Here, we consider the case where the conditional exchangeability assumption for missing data depends on more variables than age. Let W denote the vector of the following variables: gender, weight, and height. Here, we make the assumption that missing data on SBP is non-informative given age, gender, weight, and height. The revised conditional exchangeability assumption is $E[Y \mid W = w, X = x] = E[Y \mid W = w, X = x, R = 1]$ for all w in the support of W (i.e., all unique gender-weight-height combinations present in the population) and all ages x from 2-17. Similar to before, this comes along with the following positivity assumption: $\Pr(R = 1 \mid W = w, X = x) > 0$ for all w in the support of W and all ages x from 2-17. For the NHANES example, we assume that the positivity assumption holds except for children aged 2-7. This assumption matches the design of NHANES as the variables other than age did not preclude measurement of SBP. Given this exchangeability and positivity assumption, it follows that

$$E[Y] = E[E(Y \mid W, X)] = E[E(Y \mid W, X, R = 1)]$$

which is a generalization of the identification result reported in the main paper using the law of iterated expectations and exchangeability with positivity by W, X. As before, positivity presents a challenge to identification and estimation.

As in the main manuscript, $E[Y]$ can be factored into the positive and non-positive regions since $E[Y] = E[Y \mid X^* = 1] \Pr(X^* = 1) + E[Y \mid X^* = 0] \Pr(X^* = 0)$. Again, a statistical and mathematical model are considered for $E[Y \mid X^* = 1]$ and $E[Y \mid X^* = 0]$, respectively. For $E[Y \mid X^* = 1]$, we can again rely on a modified version of the prior identification result to obtain

$$E[Y \mid X^* = 1] = E[E(Y \mid W, X, X^* = 1, R = 1) \mid X^* = 1]$$

following conditional exchangeability with positivity by W, X among those 8-17 years old. This result suggests a g-computation estimator where a model for $E(Y \mid W, X, X^* = 1, R = 1)$ is fit and then used to fill-in the observations in the positive region. Unlike in the main manuscript, a saturated model is no longer as feasible to fit. Instead, a parametric model is used. Here, age is modeled using a restricted quadratic spline with knots at 10, 13, 15. Similarly, both weight and height were modeled using restricted quadratic splines with 3 knots each at 25, 35, 85 and 90, 140, 160; respectively. Models were separately estimated by gender.

For the mathematical model, no change is required in this extended version of the exchangeability assumption. By dividing the parameter into two pieces, the mathematical model solely depends on the external information available, which has not changed with the extension of exchangeability to include W for the positive region.

Therefore, the same mathematical model can be used as in the main paper, which already incorporates age, gender, and height.

For estimation with the synthesis model, the same resampling procedure described in Appendix 1 is used. For comparison with the synthesis model, we also estimated the mean SBP in children aged 8-17 accounting for informative missing by age, gender, weight, and height and extrapolating SBP for those aged 2-17. For both analyses, we drop the single additional observation with a missing value for weight.

When applying the extrapolation approach, the estimated mean SBP was 100.8 (95% CI: 97.7, 103.8). This estimate is slightly lower than the extrapolation result reported in the main paper with noticeably wider confidence intervals. The synthesis model instead estimated a mean SBP of 100.5 (95% CI: 99.9, 101.0), which matches the synthesis model results of the main paper up to the first decimal place. The synthesis model remaining similar with a change in the extrapolation approach indicates that the other variables (height, weight, gender) were not strongly related to missingness within the positive region but may have improved the extrapolations across the nonpositive region for the statistical model. Further, age was modeled more flexibly, which may have led to better agreement between models. This analysis further highlights the advantage of separating the parameter into positive and non-positive regions within the synthesis model.

Appendix 3: Alternative Synthesis Estimators for Missing Data

In this section, we consider alternative statistical models for estimation of μ. Continuing with the use of parametric models from the prior section, their use adds an additional assumption regarding correct statistical model specification.[3] G-computation relies on modeling the outcome process, but inverse probability weighting (IPW) estimators rely on statistical models for the missingness process.[4,5] Finally, augmented inverse probability weighting (AIPW) estimators use both statistical models and are doubly robust in that the estimator is consistent as long as one of two statistical models is correctly specified.[6–9] Here, we review the use of IPW and AIPW with synthesis estimators.

As in the main paper, the parameter of interest can be decomposed into

$$E[Y] = E[Y \mid X^* = 1] \Pr(X^* = 1) + E[Y \mid X^* = 0] \Pr(X^* = 0). \qquad \text{(A3.1)}$$

As before, a statistical model is used to estimate $E[Y \mid X^* = 1]$ and a mathematical model is used to estimate $E[Y \mid X^* = 0]$. In the following, we consider alternative estimators for $E[Y \mid X^* = 1]$ but the mathematical model for $E[Y \mid X^* = 0]$ remains the same since the external information is not modified.

Appendix 3.1: Inverse Probability Weighting

The IPW identification expression is

$$E[Y \mid X^* = 1] = E\left[\frac{Y\,R}{\Pr(R = 1 \mid X, W, X^* = 1)} \mid X^* = 1\right]$$

Notice that the IPW expression involves a model for the missingness process (denominator of the right-hand side) rather than the outcome process. Here, this probability can be estimated using logistic regression, or weighted logistic regression when there are sampling weights (as in the NHANES example). After estimating the probability of SBP being observed, the weighted mean of SBP among the complete cases can be computed. This estimate of $E[Y \mid X^* = 1]$ can then be combined with the mathematical model following the expression in Equation A3.1. For estimation of the uncertainty, one could then use the re-sampling procedure described in Appendix 1.

Here, the extrapolation approach is no longer straightforward to apply since the IPW estimator does not specify an outcome model from which to extrapolate. As described elsewhere,[10,11] using an IPW estimator to extrapolate requires the addition of an outcome model.

Appendix 3.2: Augmented Inverse Probability Weighting

The AIPW estimator combines g-computation and IPW estimators in such a way that if either the statistical model for the outcome process or the missingness process, but not necessarily both, is correctly specified then the AIPW estimator is consistent. Practically, this means that the AIPW estimator weakens the underlying parametric modeling assumptions relative to the g-computation and IPW estimators. There are several ways to implement AIPW,[12] but here we describe a weighted-regression implementation due to its ease of implementation.[13] To estimate $E[Y \mid X^* = 1]$, first one estimates the probability of being observed, $\Pr(R = 1 \mid X, W, X^* = 1)$, using a (weighted) logistic regression model. These probabilities are then inverted, $R / \Pr(R = 1 \mid X, W, X^* = 1)$, to construct inverse probability of missingness weights. Next, one fits a model for the outcome as done with g-computation. However, this model is now fit using weighted least squares regression with the inverse probability of missingness weights. If the study has sampling weights, this model is fit using the overall weight for an observation (computed as the product of the sampling weight and missingness weight). This fitted model can then be used following the g-computation procedure described in the main paper. This estimate of $E[Y \mid X^* = 1]$ can again be combined with the mathematical model following the expression in Equation A3.1. For estimation of the uncertainty, one can again use the re-sampling procedure described in Appendix 1.

Since an outcome model is specified, the extrapolation approach is straightforward to use with the AIPW estimator.

Appendix 3.3: Results

For the missingness model, a logistic regression model was used including main effects and splines for the continuous variables (age, height, weight). Applying the AIPW estimator for the extrapolation approach gave similar results to the extrapolation g-computation (mean: 101.1; 95% CI: 98.4, 103.8). Again, the synthesis results were unchanged for the reported number of decimals (mean: 100.5; 95% CI: 99.9, 101.0).

Appendix Table 1: Age distribution and missing systolic blood pressure ($n = 2572$)

Age	Number of Participants (%)*	Number Missing SBP (%)
2	197 (5.3%)	197 (100%)
3	157 (6.2%)	157 (100%)
4	168 (6.1%)	168 (100%)
5	166 (5.9%)	166 (100%)
6	147 (5.7%)	147 (100%)
7	153 (5.3%)	153 (100%)
8	183 (6.6%)	15 (8.1%)
9	190 (7.2%)	25 (13.2%)
10	183 (6.2%)	16 (8.7%)
11	168 (6.3%)	16 (9.5%)
12	144 (6.1%)	15 (10.4%)
13	143 (6.9%)	11 (7.7%)
14	153 (6.9%)	11 (7.2%)
15	127 (5.5%)	9 (7.1%)
16	149 (7.2%)	6 (4.0%)
17	144 (6.6%)	10 (6.9%)

SBP: systolic blood pressure.

* Sample-weighted percentage using the full sampling weights from the 2017-2018 National Health and Nutrition Examination Survey.

References

1. Kulesa A, Krzywinski M, Blainey P, Altman N. Sampling distributions and the bootstrap. *Nature Methods*. 2015;12(6):477-478. doi:10.1038/nmeth.3414

2. Ross RK, Zivich PN, Stringer JSA, Cole SR. M-estimation for common epidemiological measures: introduction and applied examples. *International Journal of Epidemiology*. 2024;53(2):dyae030. doi:10.1093/ije/dyae030

3. Aronow PM, Robins JM, Saarinen T, Sävje F, Sekhon J. Nonparametric identification is not enough, but randomized controlled trials are. *Observational Studies*. 2025;11(1):3-16.

4. Cole SR, Zivich PN, Edwards JK, et al. Missing Outcome Data in Epidemiologic Studies. *American Journal of Epidemiology*. 2023;192(1):6-10. doi:10.1093/aje/kwac179

5. Seaman SR, White IR. Review of inverse probability weighting for dealing with missing data. *Stat Methods Med Res*. 2013;22(3):278-295. doi:10.1177/0962280210395740

6. Funk MJ, Westreich D, Wiesen C, Stürmer T, Brookhart MA, Davidian M. Doubly robust estimation of causal effects. *Am J Epidemiol*. 2011;173(7):761-767. doi:10.1093/aje/kwq439

7. Bang H, Robins JM. Doubly robust estimation in missing data and causal inference models. *Biometrics*. 2005;61(4):962-973.

8. Vansteelandt S, Carpenter J, Kenward MG. Analysis of incomplete data using inverse probability weighting and doubly robust estimators. *Methodology: European Journal of Research Methods for the Behavioral and Social Sciences*. 2010;6(1):37-48. doi:10.1027/1614-2241/a000005

9. Seaman SR, Vansteelandt S. Introduction to Double Robust Methods for Incomplete Data. *Statistical Science*. 2018;33(2):184-197. doi:10.1214/18-STS647

10. Zivich PN, Edwards JK, Lofgren ET, Cole SR, Shook-Sa BE, Lessler J. Transportability without positivity: a synthesis of statistical and simulation modeling. *Epidemiology*. 2024;35(1):23-31. doi:10.1097/EDE.0000000000001677

11. Zivich PN, Edwards JK, Shook-Sa BE, Lofgren ET, Lessler J, Cole SR. Synthesis estimators for transportability with positivity violations by a continuous covariate. *Journal of the Royal Statistical Society Series A: Statistics in Society*. 2025;188(1):158-180. doi:10.1093/jrsssa/qnae084

12. Shook-Sa BE, Zivich PN, Lee C, et al. Double robust variance estimation with parametric working models. *Biometrics*. 2025;81(2):ujaf054. doi:10.1093/biomtc/ujaf054

13. Vansteelandt S, Keiding N. Invited Commentary: G-Computation–Lost in Translation? *American Journal of Epidemiology*. 2011;173(7):739-742. doi:10.1093/aje/kwq474
